# Could a Different View of Quiescence Help Us Understand How Neurogenesis Is Regulated?

**DOI:** 10.3389/fnins.2022.878875

**Published:** 2022-03-31

**Authors:** Noelia Urbán

**Affiliations:** Institute of Molecular Biotechnology of the Austrian Academy of Sciences (IMBA), Vienna BioCenter (VBC), Vienna, Austria

**Keywords:** adult neurogenesis, adult stem cells, adult neural stem cells, activation, working model, NSC transitions, dentate gyrus, subependymal zone

## Abstract

The majority of adult neural stem cells (aNSCs) are in a distinct metabolic state of reversible cell cycle exit also known as quiescence. The rate of aNSC activation determines the number of new neurons generated and directly influences the long-term maintenance of neurogenesis. Despite its relevance, it is still unclear how aNSC quiescence is regulated. Many factors contribute to this, like aNSC heterogeneity, the lack of reliable quiescence markers, the complexity of the neurogenic niches or the intricacy of the transcriptional and post-transcriptional mechanisms involved. In this perspective article I discuss possible solutions to these problems. But, first and foremost, I believe we require a model that goes beyond a simple transition toward activation. Instead, we must acknowledge the full complexity of aNSC states, which include not only activation but also differentiation and survival as behavioural outcomes. I propose a model where aNSCs dynamically transition through a cloud of highly interlinked cellular states driven by intrinsic and extrinsic cues. I also show how a new perspective enables us to integrate current results into a coherent framework leading to the formulation of new testable hypothesis. This model, like all others, is still far from perfect and will be reshaped by future findings. I believe that having a more complete view of aNSC transitions and embracing their complexity will bring us closer to understand how aNSC activity and neurogenesis are controlled throughout life.

## Introduction

**FIGURE 1:**
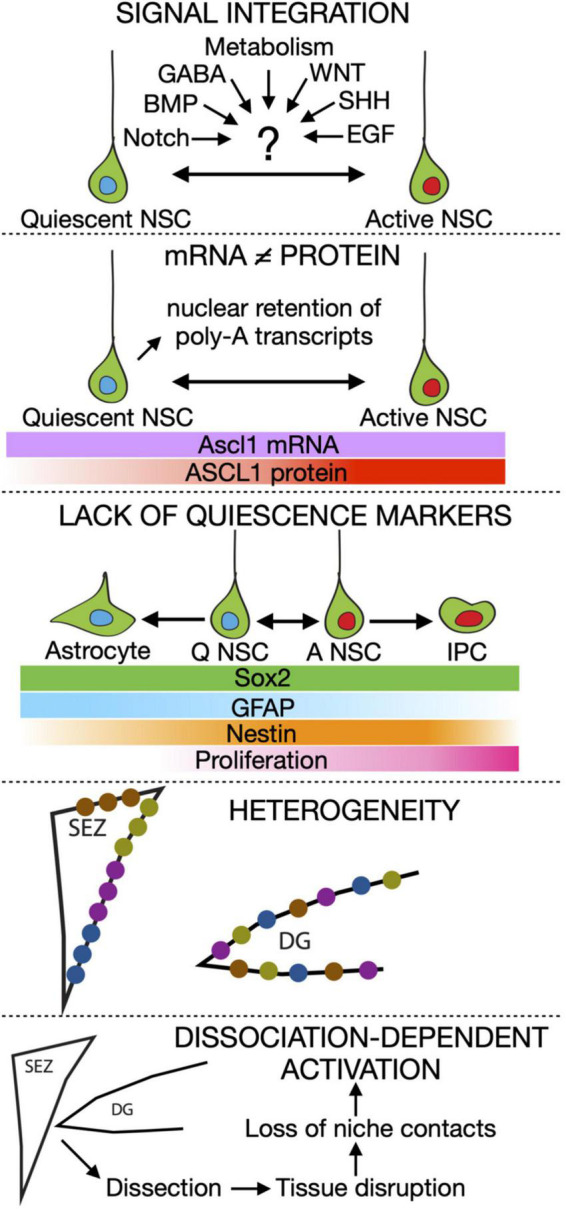
Summary of the current roadblocks in our understanding of adult neural stem cell activation. IPC, Intermediate progenitor cell.

Neurogenesis is preserved in specific regions or neurogenic niches in the adult brains of most mammals (Ming and Song, 2011; Bond et al., 2015). The subependymal zone (SEZ) of the lateral ventricles and the subgranular zone (SGZ) of the dentate gyrus (DG) in the hippocampal formation are the two main neurogenic niches in the mouse brain (Gonçalves et al., 2016; Obernier and Alvarez-Buylla, 2019). Adult neural stem cells (aNSCs) in both niches are largely quiescent (Urbán et al., 2019). Quiescence is not only the reversible exit of stem cells from the cell cycle, but also involves significant metabolic changes and the maintenance of low transcription and translation levels (Urbán and Cheung, 2021). It is hypothesised that the quiescent state protects stem cells from DNA and protein damage (Cheung and Rando, 2013). Adult NSCs have a poor ability to self-renew, and consequently, their numbers (and thus the amount of newly generated neurons) fall significantly with age in an activation-dependent way (Encinas et al., 2011; Kalamakis et al., 2019). Quiescence prevents them from being depleted, ensuring a steady supply of new cells. Therefore, the rate of aNSC activation determines the number of new neurons generated and directly influences the long-term maintenance of neurogenesis. Adult-born neurons play important functions in olfaction, memory and mood regulation, with loss of neurogenesis associated with the loss of cognitive and affective functions (Bowers and Jessberger, 2016). Despite our best efforts, we still lack a clear picture of how the transitions of aNSCs from quiescent to active states are controlled, due to several reasons (Figure 1):

- We still ignore how signal integration works in aNSCs. A variety of different signals have been involved in balancing quiescence and activation in these cells. For instance, NOTCH and BMP are pro-quiescence signals while WNT and SHH are considered pro-activation ones (Ahn and Joyner, 2005; Lie et al., 2005; Imayoshi et al., 2010; Mira et al., 2010). Metabolic cues and neurotransmitters such as GABA are also heavily involved (Alfonso et al., 2012; Berg et al., 2013; Knobloch and Jessberger, 2017; Paul et al., 2017; Catavero et al., 2018; Adusumilli et al., 2020). But in reality, cells rarely respond to a single signal, instead receiving a complex mix of signalling and metabolic cues simultaneously. The mechanisms aNSCs employ to integrate these signals and how signal dynamics influence quiescence are still poorly explored.
- Transcriptional profiling is not an accurate readout of the position of NSCs along the quiescence to activation transition. Recent data has pointed out that unlike other cell types, the correlation between mRNA and protein content in quiescent NSCs is very poor (Rossi et al., 2021). In addition, several pieces of evidence point to post-transcriptional and post-translational mechanisms being the main drivers of the transition of adult NSCs between quiescence and activation. A good example is the transcription factor ASCL1, which is essential for aNSCs to exit quiescence (Andersen et al., 2014). ASCL1 is controlled by many niche signals at different levels, from transcription to protein stability, and is therefore a perfect candidate (although likely not the only one) to integrate stimuli to make cellular fate choices. Remarkably, while ASCL1 protein is detected only in active aNSCs, Ascl1 mRNA levels are similar in quiescent and active DG aNSCs (Blomfield et al., 2019).
- Quiescence-specific markers do not exist for aNSCs. Despite many genes being enriched in quiescent NSCs compared with active NSCs, none of them has been proven to be a reliable quiescence-specific marker. The main reason for this is the huge overlap in expression profile between astrocytes and aNSCs (which are correctly called radial glia-like cells) (Urbán et al., 2019). This problem clearly demonstrates how little we know about what defines quiescence, aside from the absence of cell cycle markers.
- Adult neural stem cells are heterogeneous. SEZ NSCs are spatially heterogeneous, as they retain the regional identity that was instructed to them during development (Merkle et al., 2014; Fiorelli et al., 2015; Mizrak et al., 2019). Spatial heterogeneity has so far not been described for the DG but there, aNSCs labelled with different lineage tracing strategies show distinct activation and differentiation potential (Bonaguidi et al., 2011; Bottes et al., 2021). Not only that, but they behave differently depending on their previous activation history, with those that recently activated (resting) being much more likely to proliferate again than the ones which have remained quiescent for a long time (dormant) (Urbán et al., 2016). However, direct proof of the existence of independent lineages of DG aNSCs is still missing, as their behavioural heterogeneity has so far not been confirmed by single cell RNA sequencing (scRNAseq) data. In addition, aNSCs are heterogeneous in their response to signalling cues. In active NSCs, WNT stimulation promotes exit from the cell cycle and neuronal differentiation but the very same signal triggers proliferation in quiescent aNSCs (Austin et al., 2021). The nature and consequences of aNSC heterogeneity remain unexplored, but heterogeneity has been proposed to avoid the exhaustion of the stem cell pool upon receiving a pro-activation signal (Kalamakis et al., 2019; Martín-Suárez et al., 2019; Harris et al., 2020).
- Likely underpinning all the above, we are still lacking a true profile of NSC quiescence *in vivo*. The maintenance of quiescence depends on intrinsic as well as niche signals. The mere fact of dissociating the tissue for subsequent sorting and sequencing is enough to induce the activation of satellite cells, the resident quiescent cells of the muscle (Machado et al., 2017; van Velthoven et al., 2017). Adult NSCs are also highly dependent on niche signals and cell-cell contacts for maintaining quiescence and thus likely to suffer from similar dissociation-dependent artefacts. This could explain the high percentage of activated and primed populations of NSCs identified using RNA sequencing in comparison to data obtained through immunohistochemistry.

## Some Steps to Move Forward

The points above show that improved methods are needed to reliably detect the steps followed by adult NSCs as they exit quiescence and the mechanisms that drive their switch to activation.

Even though mRNA content might not be the biggest determinant of aNSC fate, heterogeneity is noticeable in published scRNAseq data in both SEZ and DG NSCs (Llorens-Bobadilla et al., 2015; Shin et al., 2015; Artegiani et al., 2017; Dulken et al., 2017; Basak et al., 2018; Hochgerner et al., 2018). But in order to capture the true signature of quiescent NSCs we must make sure to avoid dissociation artefacts. One way to do so would be to perform spatial transcriptomics on brain samples. However, even with the latest advances, the sequencing depth and cellular resolution of this technique is not yet enough for assessing differences between aNSCs. Alternatively, we could adapt to the brain niches the method that was used to solve the very same problem in satellite stem cells. By mildly perfusing the mice prior to tissue dissection we should be able to lock NSCs states while preserving RNA of enough quality for subsequent sequencing (Yue and Cheung, 2020).

But since changes in signalling and protein stability are crucial for aNSC decisions, we cannot rely solely on transcriptional data. One way to complement transcriptional approaches is by multiplexed immunohistochemistry, with which it will be possible to identify stem cells along with signalling and cell fate readouts (Cole et al., 2022). One immediate caveat of this technique is that it relies on the quality of the available antibodies, which is often sub-optimal for phospho-proteins and other signalling-related post-transcriptional modifications (PTMs). It is therefore necessary to generate reporter lines to closely monitor signalling pathway activation along aNSC transitions.

Ultimately, we will need proof that given signalling or metabolic cues are responsible for the transition of aNSCs through different states. For this, we must be able to manipulate those cues on aNSCs and evaluate their functional outcome. The scarcity of aNSCs, the lack of reliable unique markers for them and the complexity of their surrounding niches make such an approach extremely challenging *in vivo*. This problem can be overcome by using a simplified, controlled *in vitro* system. Adult NSCs can be isolated from both the SEZ and DG and remain in a proliferative, undifferentiated state for many passages in culture. Upon addition of BMP4, known to maintain quiescence *in vivo*, the cells enter a reversible quiescence-like state. The system, first described by Mira et al. (2010) and further developed by many other groups, recapitulates important aspects of quiescence, including withdrawal from the cell cycle, upregulation of astroglial markers and profound metabolic changes (Mira et al., 2010; Martynoga et al., 2013; Knobloch et al., 2017; Leeman et al., 2018). The simplicity of the system allows to directly measure the effects of signalling and metabolic cues on aNSC behaviour.

Last but not least, in order to integrate recent discoveries on the tremendous complexity of quiescence regulation in adult NSCs, we need a new framework that goes beyond our existing concept of a linear shift from quiescence to activation.

## A Full, Dynamic View of Quiescence

Single cell RNA sequencing changed our view from discrete active or quiescent populations of aNSCs to a continuous transition of states (Urbán et al., 2019). Pseudo-time analysis then helped streamline the data and focus the attention on those genes expressed highest or lowest in quiescence and which are down or upregulated, respectively, during activation (Shin et al., 2015; Dulken et al., 2017). Different degrees of quiescence have been identified, depending on how close the transcriptional profile is to the deepest or shallowest end of the quiescence-to-activation spectrum (deep, dormant, primed, active, etc.). While this has been extremely helpful, it has also oversimplified our view of quiescence.

**FIGURE 2:**
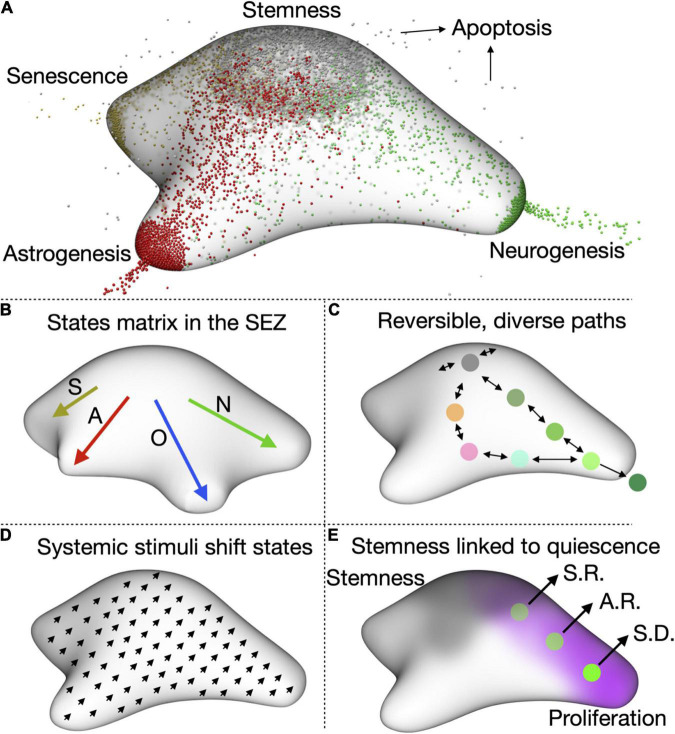
A complete view of aNSC quiescence. **(A)** Representation of the states matrix for the DG showing the four main outlets, neurogenesis, astrogenesis, senescence, and apoptosis. Their relative weight depends on the age and condition (e.g., stress levels, disease, etc.) of the animal. **(B)** Representation of the states matrix for the SEZ, in which oligodendrogenesis is also an important outlet. S, senescence; A, astrogenesis; O, oligodendrogenesis; N, neurogenesis. **(C)** cell can reversibly transition through the matrix space and several paths are possible for the same outcome (in this case, neurogenesis). **(D)** One of our hypotheses is that systemic stimuli, such as diet of exercise, will shift the cells in the matrix to a slightly different state. This could then affect how they respond to additional stimuli. **(E)** We also notice that proliferation is inversely correlated with stemness, which suggests that quiescence and stemness could be functionally linked. aNSCs lose self-renewing potential as they continue proliferating. S.R.: symmetric self-renewal, A.R.: asymmetric self-renewal, S.D.: symmetric differentiation.

To fully understand the quiescence to activation transitions of aNSCs (and in fact any cellular transition), we must embrace their complexity in full. Therefore, instead of the classical view of a linear transition from quiescence to activation, I propose a model where adult NSCs exist in a vast matrix of cellular states (Figure 2). These states are not restricted to a switch between activation and quiescence, but also involve other stem cell characteristics. In this complete view, NSCs transit through a wide spectrum of states which differ in their differentiation, proliferation, metabolic, fitness and survival capabilities. In the case of adult NSCs, the drains (i.e., the ways in which NSCs stop being stem cells) are four: neurogenesis, gliogenesis, senescence and apoptosis. For NSCs in the DG, neurogenesis is the one with the most weight, as gliogenesis (mostly astrogliogenesis) happens rarely (Figure 2A). For the SEZ, both gliogenesis (preferentially oligodendrogenesis) and neurogenesis constitute important drains (Figure 2B). In both niches, apoptosis of NSCs is relatively low and senescence gains importance as ageing progresses.

At one specific time each NSC cell occupies a defined position in the states matrix. But if we were able to follow that same NSC over time we would be able to observe it travelling (or reversibly transitioning) through different positions (Figure 2C). NSCs in opposite sides of the matrix can present very different transcriptional profiles, with cells leaning toward activation expressing cell cycle genes and those close to the astrogliogenic output expressing astrocyte-enriched genes. ScRNAseq data could therefore be used as a starting point to roughly position cells in the matrix and identify the factors and cues driving NSC behaviour. Provided sequencing data is of enough quality and depth, we could even gain insight into the intrinsic and niche-related differences between SEZ and DG and identify age-related changes in NSC states. This has already been possible with current sequencing pipelines and should be very much improved when paired to techniques aimed at conserving the quiescence signature *in vivo* (Dulken et al., 2017; Kalamakis et al., 2019; Mizrak et al., 2019; Borrett et al., 2020, 2022). The use of tools such RNAvelocity (La Manno et al., 2018) and CellRank (Lange et al., 2022) on scRNAseq data will allow us to measure the direction and speed of cells travelling through the matrix and help us identify regions where one direction is favoured (where the probability of going back to the previous state is much lower than that of continuing in the same direction).

However at shorter distances, which are those important for the actual behavioural decisions of aNSCs, transcriptional differences can be small, noisy and, I would argue, rather meaningless. The main drivers of the movement of adult NSCs through different states are signalling cues and the activity of intrinsic factors. To fully understand how NSCs navigate through these spaces, the focus of future research should lay on signal integration, protein stability and PTMs. One way to integrate signalling activity with aNSC behavioural output would be to focus on known direct readouts of signalling pathways (through direct detection or the use of reporters) and correlate them with stemness, proliferation, differentiation, cell death and senescence markers. This, while extremely challenging *in vivo*, would be fully possible to achieve thanks to the *in vitro* system. *In vitro* aNSC states exist in a fully defined and stable environment and are thus unlikely to be common (or even exist) *in vivo*. Direct manipulation of signalling and metabolism in aNSC *in vitro* will also allow evaluating the effects of single and combined cues on the transition of aNSCs between states. In addition, the *in vitro* system will enable us to determine the degree of intrinsic heterogeneity of adult NSCs in different conditions. Intrinsic heterogeneity can be driven, for instance, by oscillations in the levels of key transcription factors [ASCL1 oscillates in embryonic NSCs (Imayoshi et al., 2013)] and the rhythmicity of signalling cascades (like NOTCH or WNT). Cyclic expression of fate determinants belonging to gene regulatory networks has been suggested to underlie the multipotency of neural crest and neural stem cells (Imayoshi and Kageyama, 2014; Kelsh et al., 2021).

## Revising Our View of Current Data and Formulating New Hypotheses

The new framework immediately prompts us to think of the heterogeneity of stem cells in a different way and allows the integration of clonal and label-retention data without the need of invoking the existence of distinct stem cell types. The clonal approaches typically used for the study of adult neurogenesis label those cells with the highest activity of a particular promoter at the time of tamoxifen administration. They will therefore label the positions in the matrix where that promoter is most active. But labelled cells might not constitute a separate lineage, as they do not necessarily need to be restricted to one single area of the matrix and could partially overlap with the high activity of other promoters also used for clonal analysis. For instance, high activity of the Gli1 promoter could happen anywhere where SHH signalling is high and is likely to overlap, at least partially, with high expression of unrelated genes such as Nestin or Ascl1. It is important to note that promoter activity and gene function do not always come hand in hand. Even less in adult NSCs, where mRNA and protein are poorly correlated. Nevertheless, the observed behavioural biases could still be linked to the function of the gene used for labelling the cells. For instance, high Ascl1 mRNA expression, although does not always lead to activation due to post-transcriptional regulation, could increase the probability of NSCs to activate. On the other hand, it is easy to imagine that resting NSCs, which recently proliferated, are likely to remain in a pro-activation zone after division. This alone will make them more likely to become activated again than dormant NSCs, which did not activate during the labelling period. Finally, cells which are very distant in the matrix might have very distinct signal transduction capabilities, both due to differential expression of signalling pathway components and different metabolic characteristics. This readily explains how NSCs respond differently to the same stimuli depending on their state (e.g., response to WNT in active vs. quiescent cells).

The model makes it clear that the likeliness of a NSC becoming activated depends on two things: their current state (the closer to the activation zone, the most likely it is that they will become activated), and their signalling environment (how likely it is that they will receive the appropriate activation signals). But what I personally find most exciting about this framework is that it allows us to generate hypothesis and design specific experiments to test them. Two particularly tantalising ones are:

### Do Systemic Cues Shift Adult Neural Stem Cells States, Affecting Their Response to Further Signals?

One still unanswered question in the field is how interventions that affect neurogenesis impact the maintenance of aNSCs over long periods of time. Stimuli such as exercise, dietary changes or an enriched environment affect adult neurogenesis (Trejo et al., 2001; Overall et al., 2016; Kempermann, 2019; Dias et al., 2021). However, virtually nothing is known about their long-term influence on NSC quiescence. In the light of the new model, I propose that these stimuli shift aNSCs to a different state, changing the whole range of aNSC states (Figure 2D). This resembles how ageing pushes aNSCs toward senescence, injury toward activation or epilepsy toward astrogenesis. Indeed, the global changes in aNSC behaviour over time suggest that ageing heavily influences the positioning of aNSC in the matrix (Kalamakis et al., 2019; Martín-Suárez et al., 2019; Harris et al., 2020). For more subtle stimuli, the shift might not result in an obvious, immediate phenotype. But it will affect the response of NSCs to additional signals and systemic cues, including ageing. Evaluating potential effects on quiescence is of particular importance for interventions suggested to counteract ageing, as we risk generating a brief burst in neurogenesis at the cost of exhausting aNSCs at an even faster pace.

### Is Quiescence Intimately Linked to Stemness?

Several pieces of independent evidence suggest that quiescence does not serve the sole purpose of pausing the cell cycle. Stem cell-related markers (e.g., Sox9, Hopx) are highest in the most quiescent cells and repeated activation of DG NSCs *in vivo* is associated with the loss of their self-renewing ability (Llorens-Bobadilla et al., 2015; Shin et al., 2015; Pilz et al., 2018; Bottes et al., 2021). This suggests that quiescence is important for the self-renewal capacity of adult NSCs (Figure 2E). Such a connection is evident from our model, which also points that in order to measure it, we need to go beyond merely assessing proliferation levels and jointly analyse fitness and self-renewal capacity of adult NSCs as they transition from quiescent to active states. Determining if and how quiescence and self-renewal are linked will help devise better strategies to avoid NSC exhaustion while still generating enough neurons to maintain functionality throughout life.

## Conclusion and Future Perspectives

The way we think about a subject often affects the questions we ask and due to the oversimplification associated with models, significant features could be overlooked. I believe it is time to incorporate the intricacy of stem cell behaviour into our working models, even if we still do not have enough data to fully support them. In fact, this, as any model is only meant to serve as a starting point, since it will continuously evolve through the addition of new data. Experiments, on the other hand, are independent of the model and will remain significant regardless of it. Although my focus is on aNSCs, the proposed framework will be useful for other adult and embryonic stem cell transitions, reprogramming strategies and cancer research. One interesting avenue would be to further explore the continuum of states that exist between glia and neural stem cells. Glial cells respond to injury and have the potential to generate new neurons in the adult brain. By investigating the aNSC to glia transition as well as the reactivation path of glial cells, we could identify important roadblocks for glia-to-neuron transitions (Gascón et al., 2017). Other systems, particularly simpler models, could teach us a lot about the basic principles governing cell fate transitions. Similarly, looking at other cells in the niche will provide a more complete picture of aNSC regulation and help us understand the significance of cell-cell interactions. Through the combination of new approaches and this framework we may soon be able to understand the rules of the game and make sense of the apparent stochasticity of aNSC activation.

## Data Availability Statement

The original contributions presented in the study are included in the article/supplementary material, further inquiries can be directed to the corresponding author.

## Author Contributions

NU conceived and wrote the manuscript.

## Conflict of Interest

The author declares that the research was conducted in the absence of any commercial or financial relationships that could be construed as a potential conflict of interest. The reviewer JE-P declared a past co-authorship with the author NU to the handling editor.

## Publisher’s Note

All claims expressed in this article are solely those of the authors and do not necessarily represent those of their affiliated organizations, or those of the publisher, the editors and the reviewers. Any product that may be evaluated in this article, or claim that may be made by its manufacturer, is not guaranteed or endorsed by the publisher.

## References

[B1] Adusumilli V. S. Walker T. L. Overall R. W. Klatt G. M. Zeidan S. A. Zocher S. (2020). ROS Dynamics Delineate Functional States of Hippocampal Neural Stem Cells and Link to Their Activity-Dependent Exit from Quiescence. *Cell Stem Cell* 28 300–314.e6. 10.1016/j.stem.2020.10.019 33275875 PMC7875116

[B2] Ahn S. Joyner A. L. (2005). In vivo analysis of quiescent adult neural stem cells responding to Sonic hedgehog. *Nature* 437 894–897. 10.1038/nature03994 16208373

[B3] Alfonso J. Le Magueresse C. Zuccotti A. Khodosevich K. Monyer H. (2012). Diazepam binding inhibitor promotes progenitor proliferation in the postnatal SVZ by reducing GABA signaling. *Cell Stem Cell* 10 76–87. 10.1016/j.stem.2011.11.011 22226357

[B4] Andersen J. Urbán N. Achimastou A. Ito A. Simic M. Ullom K. (2014). A transcriptional mechanism integrating inputs from extracellular signals to activate hippocampal stem cells. *Neuron* 83 1085–1097. 10.1016/j.neuron.2014.08.004 25189209 PMC4157576

[B5] Artegiani B. Lyubimova A. Muraro M. van Es J. H. van Oudenaarden A. Clevers H. (2017). A Single-Cell RNA Sequencing Study Reveals Cellular and Molecular Dynamics of the Hippocampal Neurogenic Niche. *Cell Rep.* 21 3271–3284. 10.1016/j.celrep.2017.11.050 29241552

[B6] Austin S. H. L. Gabarró-Solanas R. Rigo P. Paun O. Harris L. Guillemot F. (2021). Wnt/β-catenin signalling is dispensable for adult neural stem cell homeostasis and activation. *Development* 148:dev199629. 10.1242/dev.199629 34557919 PMC8572000

[B7] Basak O. Krieger T. G. Muraro M. J. Wiebrands K. Stange D. E. Frias-Aldeguer J. (2018). Troy+ brain stem cells cycle through quiescence and regulate their number by sensing niche occupancy. *Proc. Natl. Acad. Sci. U. S. A.* 115 E610–E619. 10.1073/pnas.1715911114 29311336 PMC5789932

[B8] Berg D. A. Belnoue L. Song H. Simon A. (2013). Neurotransmitter-mediated control of neurogenesis in the adult vertebrate brain. *Development* 140 2548–2561. 10.1242/dev.088005 23715548 PMC3666382

[B9] Blomfield I. M. Rocamonde B. Masdeu M. D. M. Mulugeta E. Vaga S. van den Berg D. L. (2019). Id4 promotes the elimination of the pro-activation factor Ascl1 to maintain quiescence of adult hippocampal stem cells. *Elife* 8:e48561. 10.7554/eLife.48561 31552825 PMC6805120

[B10] Bonaguidi M. A. Wheeler M. A. Shapiro J. S. Stadel R. P. Sun G. J. Ming G. (2011). In vivo clonal analysis reveals self-renewing and multipotent adult neural stem cell characteristics. *Cell* 145 1142–1155. 10.1016/j.cell.2011.05.024 21664664 PMC3124562

[B11] Bond A. M. Ming G.-L. Song H. (2015). Adult Mammalian Neural Stem Cells and Neurogenesis: five Decades Later. *Cell Stem Cell* 17 385–395. 10.1016/j.stem.2015.09.003 26431181 PMC4683085

[B12] Borrett M. J. Innes B. T. Jeong D. Tahmasian N. Storer M. A. Bader G. D. (2020). Single-Cell Profiling Shows Murine Forebrain Neural Stem Cells Reacquire a Developmental State when Activated for Adult Neurogenesis. *Cell Rep.* 32:108022. 10.1016/j.celrep.2020.108022 32783944

[B13] Borrett M. J. Innes B. T. Tahmasian N. Bader G. D. Kaplan D. R. Miller F. D. (2022). A Shared Transcriptional Identity for Forebrain and Dentate Gyrus Neural Stem Cells from Embryogenesis to Adulthood. *Eneuro* 9 ENEURO.0271–21.2021. 10.1523/ENEURO.0271-21.2021 35027446 PMC8856713

[B14] Bottes S. Jaeger B. N. Pilz G.-A. Jörg D. J. Cole J. D. Kruse M. (2021). Long-term self-renewing stem cells in the adult mouse hippocampus identified by intravital imaging. *Nat. Neurosci.* 24 225–233. 10.1038/s41593-020-00759-4 33349709 PMC7116750

[B15] Bowers M. Jessberger S. (2016). Linking adult hippocampal neurogenesis with human physiology and disease. *Dev. Dyn.* 245 702–709. 10.1002/dvdy.24396 26890418

[B16] Catavero C. Bao H. Song J. (2018). Neural mechanisms underlying GABAergic regulation of adult hippocampal neurogenesis. *Cell Tissue Res.* 371 33–46. 10.1007/s00441-017-2668-y 28948349 PMC5750064

[B17] Cheung T. H. Rando T. A. (2013). Molecular regulation of stem cell quiescence. *Nat. Rev. Mol. Cell. Biol.* 14 329–340. 10.1038/nrm3591 23698583 PMC3808888

[B18] Cole J. D. Sarabia Del Castillo J. Gut G. Gonzalez-Bohorquez D. Pelkmans L. Jessberger S. (2022). Characterization of the neurogenic niche in the aging dentate gyrus using iterative immunofluorescence imaging. *Elife* 11:e68000. 10.7554/eLife.68000 35089129 PMC8798039

[B19] Dias G. P. Murphy T. Stangl D. Ahmet S. Morisse B. Nix A. (2021). Intermittent fasting enhances long-term memory consolidation, adult hippocampal neurogenesis, and expression of longevity gene Klotho. *Mol. Psychiatry* 26 6365–6379. 10.1038/s41380-021-01102-4 34031536 PMC8760057

[B20] Dulken B. W. Leeman D. S. Boutet S. C. Hebestreit K. Brunet A. (2017). Single-Cell Transcriptomic Analysis Defines Heterogeneity and Transcriptional Dynamics in the Adult Neural Stem Cell Lineage. *Cell Rep.* 18 777–790. 10.1016/j.celrep.2016.12.060 28099854 PMC5269583

[B21] Encinas J. M. Michurina T. V. Peunova N. Park J.-H. Tordo J. Peterson D. A. (2011). Division-Coupled Astrocytic Differentiation and Age-Related Depletion of Neural Stem Cells in the Adult Hippocampus. *Cell Stem Cell* 8 566–579. 10.1016/j.stem.2011.03.010 21549330 PMC3286186

[B22] Fiorelli R. Azim K. Fischer B. Raineteau O. (2015). Adding a spatial dimension to postnatal ventricular-subventricular zone neurogenesis. *Development* 142 2109–2120. 10.1242/dev.119966 26081572

[B23] Gascón S. Masserdotti G. Russo G. L. Götz M. (2017). Direct Neuronal Reprogramming: achievements, Hurdles, and New Roads to Success. *Cell Stem Cell* 21 18–34. 10.1016/j.stem.2017.06.011 28686866

[B24] Gonçalves J. T. Schafer S. T. Gage F. H. (2016). Adult Neurogenesis in the Hippocampus: from Stem Cells to Behavior. *Cell* 167 897–914. 10.1016/j.cell.2016.10.021 27814520

[B25] Harris L. Rigo P. Stiehl T. Gaber Z. Austin S. H. L. Masdeu M. (2020). Progressive changes in hippocampal stem cell properties ensure lifelong neurogenesis. *Biorxiv* 10.1101/2020.03.12.987107 PMC8110946 33581058

[B26] Hochgerner H. Zeisel A. Lönnerberg P. Linnarsson S. (2018). Conserved properties of dentate gyrus neurogenesis across postnatal development revealed by single-cell RNA sequencing. *Nat. Neurosci.* 21 290–299. 10.1038/s41593-017-0056-2 29335606

[B27] Imayoshi I. Isomura A. Harima Y. Kawaguchi K. Kori H. Miyachi H. (2013). Oscillatory control of factors determining multipotency and fate in mouse neural progenitors. *Science* 342 1203–1208. 10.1126/science.1242366 24179156

[B28] Imayoshi I. Kageyama R. (2014). Oscillatory control of bHLH factors in neural progenitors. *Trends Neurosci.* 37 531–538. 10.1016/j.tins.2014.07.006 25149265

[B29] Imayoshi I. Sakamoto M. Yamaguchi M. Mori K. Kageyama R. (2010). Essential roles of Notch signaling in maintenance of neural stem cells in developing and adult brains. *J. Neurosci.* 30 3489–3498. 10.1523/JNEUROSCI.4987-09.2010 20203209 PMC6634119

[B30] Kalamakis G. Brüne D. Ravichandran S. Bolz J. Fan W. Ziebell F. (2019). Quiescence Modulates Stem Cell Maintenance and Regenerative Capacity in the Aging Brain. *Cell* 176 1407–1419.e14. 10.1016/j.cell.2019.01.040 30827680

[B31] Kelsh R. N. Camargo Sosa K. Farjami S. Makeev V. Dawes J. H. P. Rocco A. (2021). Cyclical fate restriction: a new view of neural crest cell fate specification. *Development* 148:dev176057. 10.1242/dev.176057 35020872

[B32] Kempermann G. (2019). Environmental enrichment, new neurons and the neurobiology of individuality. *Nat. Rev. Neurosci.* 20 235–245. 10.1038/s41583-019-0120-x 30723309

[B33] Knobloch M. Jessberger S. (2017). Metabolism and neurogenesis. *Curr. Opin. Neurobiol.* 42 45–52. 10.1016/j.conb.2016.11.006 27915086

[B34] Knobloch M. Pilz G.-A. Ghesquière B. Kovacs W. J. Wegleiter T. Moore D. L. (2017). A Fatty Acid Oxidation-Dependent Metabolic Shift Regulates Adult Neural Stem Cell Activity. *Cell Rep.* 20 2144–2155. 10.1016/j.celrep.2017.08.029 28854364 PMC5583518

[B35] La Manno G. Soldatov R. Zeisel A. Braun E. Hochgerner H. Petukhov V. (2018). RNA velocity of single cells. *Nature* 560 494–498. 10.1038/s41586-018-0414-6 30089906 PMC6130801

[B36] Lange M. Bergen V. Klein M. Setty M. Reuter B. Bakhti M. (2022). CellRank for directed single-cell fate mapping. *Nat. Methods* 19 159–170. 10.1038/s41592-021-01346-6 35027767 PMC8828480

[B37] Leeman D. S. Hebestreit K. Ruetz T. Webb A. E. McKay A. Pollina E. A. (2018). Lysosome activation clears aggregates and enhances quiescent neural stem cell activation during aging. *Science* 359 1277–1283. 10.1126/science.aag3048 29590078 PMC5915358

[B38] Lie D.-C. Colamarino S. A. Song H.-J. Désiré L. Mira H. Consiglio A. (2005). Wnt signalling regulates adult hippocampal neurogenesis. *Nature* 437 1370–1375. 10.1038/nature04108 16251967

[B39] Llorens-Bobadilla E. Zhao S. Baser A. Saiz-Castro G. Zwadlo K. Martin-Villalba A. (2015). Single-Cell Transcriptomics Reveals a Population of Dormant Neural Stem Cells that Become Activated upon Brain Injury. *Cell Stem Cell* 17 329–340. 10.1016/j.stem.2015.07.002 26235341

[B40] Machado L. Esteves de Lima J. Fabre O. Proux C. Legendre R. Szegedi A. (2017). In Situ Fixation Redefines Quiescence and Early Activation of Skeletal Muscle Stem Cells. *Cell Rep.* 21 1982–1993. 10.1016/j.celrep.2017.10.080 29141227

[B41] Martín-Suárez S. Valero J. Muro-García T. Encinas J. M. (2019). Phenotypical and functional heterogeneity of neural stem cells in the aged hippocampus. *Aging Cell* 18:e12958. 10.1111/acel.12958 30989815 PMC6612636

[B42] Martynoga B. Mateo J. L. Zhou B. Andersen J. Achimastou A. Urbán N. (2013). Epigenomic enhancer annotation reveals a key role for NFIX in neural stem cell quiescence. *Genes Dev.* 27 1769–1786. 10.1101/gad.216804.113 23964093 PMC3759694

[B43] Merkle F. T. Fuentealba L. C. Sanders T. A. Magno L. Kessaris N. Alvarez-Buylla A. (2014). Adult neural stem cells in distinct microdomains generate previously unknown interneuron types. *Nat. Neurosci.* 17 207–214. 10.1038/nn.3610 24362763 PMC4100623

[B44] Ming G. Song H. (2011). Adult Neurogenesis in the Mammalian Brain: significant Answers and Significant Questions. *Neuron* 70 687–702. 10.1016/j.neuron.2011.05.001 21609825 PMC3106107

[B45] Mira H. Andreu Z. Suh H. Lie D. C. Jessberger S. Consiglio A. (2010). Signaling through BMPR-IA Regulates Quiescence and Long-Term Activity of Neural Stem Cells in the Adult Hippocampus. *Cell Stem Cell* 7 78–89. 10.1016/j.stem.2010.04.016 20621052

[B46] Mizrak D. Levitin H. M. Delgado A. C. Crotet V. Yuan J. Chaker Z. (2019). Single-Cell Analysis of Regional Differences in Adult V-SVZ Neural Stem Cell Lineages. *Cell Rep.* 26 394–406.e5. 10.1016/j.celrep.2018.12.044 30625322 PMC6368857

[B47] Obernier K. Alvarez-Buylla A. (2019). Neural stem cells: origin, heterogeneity and regulation in the adult mammalian brain. *Development* 146:dev156059. 10.1242/dev.156059 30777863 PMC6398449

[B48] Overall R. W. Walker T. L. Fischer T. J. Brandt M. D. Kempermann G. (2016). Different Mechanisms Must Be Considered to Explain the Increase in Hippocampal Neural Precursor Cell Proliferation by Physical Activity. *Front. Neurosci.* 10:362. 10.3389/fnins.2016.00362 27536215 PMC4971098

[B49] Paul A. Chaker Z. Doetsch F. (2017). Hypothalamic regulation of regionally distinct adult neural stem cells and neurogenesis. *Science* 356 1383–1386. 10.1126/science.aal3839 28619719

[B50] Pilz G.-A. Bottes S. Betizeau M. Jörg D. J. Carta S. Simons B. D. (2018). Live imaging of neurogenesis in the adult mouse hippocampus. *Science* 359 658–662. 10.1126/science.aao5056 29439238 PMC6986926

[B51] Rossi A. Coum A. Madelenat M. Harris L. Miedzik A. Strohbuecker S. (2021). Neural stem cells alter nucleocytoplasmic partitioning and accumulate nuclear polyadenylated transcripts during quiescence. *Biorxiv* 10.1101/2021.01.06.425462

[B52] Shin J. Berg D. A. Zhu Y. Shin J. Y. Song J. Bonaguidi M. A. (2015). Single-Cell RNA-Seq with Waterfall Reveals Molecular Cascades underlying Adult Neurogenesis. *Cell Stem Cell* 17 360–372. 10.1016/j.stem.2015.07.013 26299571 PMC8638014

[B53] Trejo J. L. Carro E. Torres-Alemán I. (2001). Circulating Insulin-Like Growth Factor I Mediates Exercise-Induced Increases in the Number of New Neurons in the Adult Hippocampus. *J. Neurosci.* 21 1628–1634. 10.1523/JNEUROSCI.21-05-01628.2001 11222653 PMC6762955

[B54] Urbán N. Blomfield I. M. Guillemot F. (2019). Quiescence of Adult Mammalian Neural Stem Cells: a Highly Regulated Rest. *Neuron* 104 834–848. 10.1016/j.neuron.2019.09.026 31805262

[B55] Urbán N. Cheung T. H. (2021). Stem cell quiescence: the challenging path to activation. *Development* 148 dev165084. 10.1242/dev.165084 33558315 PMC7888710

[B56] Urbán N. van den Berg D. L. C. Forget A. Andersen J. Demmers J. A. A. Hunt C. (2016). Return to quiescence of mouse neural stem cells by degradation of a proactivation protein. *Science* 353 292–295. 10.1126/science.aaf4802 27418510 PMC5321528

[B57] van Velthoven C. T. J. de Morree A. Egner I. M. Brett J. O. Rando T. A. (2017). Transcriptional Profiling of Quiescent Muscle Stem Cells In Vivo. *Cell Rep.* 21 1994–2004. 10.1016/j.celrep.2017.10.037 29141228 PMC5711481

[B58] Yue L. Cheung T. H. (2020). Protocol for Isolation and Characterization of In Situ Fixed Quiescent Muscle Stem Cells. *Star Protocols* 1:100128. 10.1016/j.xpro.2020.100128 33377022 PMC7757111

